# Bosutinib Therapy Ameliorates Lung Inflammation and Fibrosis in Experimental Silicosis

**DOI:** 10.3389/fphys.2017.00159

**Published:** 2017-03-15

**Authors:** Priscila J. Carneiro, Amanda L. Clevelario, Gisele A. Padilha, Johnatas D. Silva, Jamil Z. Kitoko, Priscilla C. Olsen, Vera L. Capelozzi, Patricia R. M. Rocco, Fernanda F. Cruz

**Affiliations:** ^1^Laboratory of Pulmonary Investigation, Carlos Chagas Filho Institute of Biophysics, Federal University of Rio de JaneiroRio de Janeiro, Brazil; ^2^Laboratory of Clinical Bacteriology and Immunology, Department of Toxicological and Clinical Analysis, School of Pharmacy, Federal University of Rio de JaneiroRio de Janeiro, Brazil; ^3^Laboratory of Pulmonary Genomics, Department of Pathology, School of Medicine, University of São PauloSão Paulo, Brazil

**Keywords:** tyrosine kinase inhibitor, lung fibrosis, macrophage polarization, regulatory T cells, lymphocytes, lung mechanics

## Abstract

Silicosis is an occupational lung disease for which no effective therapy exists. We hypothesized that bosutinib, a tyrosine kinase inhibitor, might ameliorate inflammatory responses, attenuate pulmonary fibrosis, and thus improve lung function in experimental silicosis. For this purpose, we investigated the potential efficacy of bosutinib in the treatment of experimental silicosis induced in C57BL/6 mice by intratracheal administration of silica particles. After 15 days, once disease was established, animals were randomly assigned to receive DMSO or bosutinib (1 mg/kg/dose in 0.1 mL 1% DMSO) by oral gavage, twice daily for 14 days. On day 30, lung mechanics and morphometry, total and differential cell count in alveolar septa and granuloma, levels of interleukin (IL)-1β, tumor necrosis factor (TNF)-α, interferon (IFN)-γ, IL-4, transforming growth factor (TGF)-β, and vascular endothelial growth factor in lung homogenate, M1 and M2 macrophages, total leukocytes, and T cells in BALF, lymph nodes, and thymus, and collagen fiber content in alveolar septa and granuloma were analyzed. In a separate *in vitro* experiment, RAW264.7 macrophages were exposed to silica particles in the presence or absence of bosutinib. After 24 h, gene expressions of arginase-1, IL-10, IL-12, inducible nitric oxide synthase (iNOS), metalloproteinase (MMP)-9, tissue inhibitor of metalloproteinase (TIMP)-1, and caspase-3 were evaluated. *In vivo*, in silicotic animals, bosutinib, compared to DMSO, decreased: (1) fraction area of collapsed alveoli, (2) size and number of granulomas, and mononuclear cell granuloma infiltration; (3) IL-1β, TNF-α, IFN-γ, and TGF-β levels in lung homogenates, (4) collagen fiber content in lung parenchyma, and (5) viscoelastic pressure and static lung elastance. Bosutinib also reduced M1 cell counts while increasing M2 macrophage population in both lung parenchyma and granulomas. Total leukocyte, regulatory T, CD4^+^, and CD8^+^ cell counts in the lung-draining lymph nodes also decreased with bosutinib therapy without affecting thymus cellularity. *In vitro*, bosutinib led to a decrease in IL-12 and iNOS and increase in IL-10, arginase-1, MMP-9, and TIMP-1. In conclusion, in the current model of silicosis, bosutinib therapy yielded beneficial effects on lung inflammation and remodeling, therefore resulting in lung mechanics improvement. Bosutinib may hold promise for silicosis; however, further studies are required.

## Introduction

Silicosis is a chronic occupational pulmonary disease caused by long-term inhalation of silica dust (Laney and Attfield, 2010; Suarthana et al., 2011; Lopes-Pacheco et al., 2014). Once these particles are inhaled, they can trigger persistent inflammation of the alveoli and further pulmonary fibrosis, resulting in a cycle of lung damage (Thakur et al., 2009). Silica particles induce macrophage activation, with consequent recruitment of lymphocytes and neutrophils, as well as fibroblasts, which are implicated in progressive tissue fibrosis (Borges et al., 2001; Rimal et al., 2005; Greenberg et al., 2007). The injury is usually irreversible and disabling, due to the formation of granulomas that reduce the area available for gas exchange and thus impair lung function (Leung et al., 2012; Kawasaki, 2015; Cruz et al., 2016). Silicosis remains a prevalent health problem worldwide, particularly in developing nations (Craighead et al., 1988; Leung et al., 2012). To date, no conventional therapy is able to alter the progressive course of the disease or reverse lung fibrosis effectively.

The Src family of tyrosine kinases (TKs) has been shown to play key roles in oxidant-mediated signal transduction in macrophages (Khadaroo et al., 2003; Kang et al., 2006) and lymphocytes (Salmond et al., 2009). These kinases interact with various signal transduction pathways downstream of different surface receptors, coordinating critical cellular processes such as proliferation and differentiation, cell survival and metabolism, cell migration, apoptosis, cytoskeletal rearrangement, and immune response (Thomas and Brugge, 1997; Okutani et al., 2006; Ahluwalia et al., 2010). Thus, they play an important regulatory role in almost all aspects of cell biology (Manning et al., 2002). Activation of protein kinases is known to occur in a variety of diseases, including cancer, inflammatory disorders (Summy and Gallick, 2003; Johnson and Gallick, 2007; Hu et al., 2008; Li, 2008; Zarbock and Ley, 2011; Liu et al., 2014; Kong et al., 2015; Ozanne et al., 2015), and fibrotic processes (Chaudhary et al., 2007; Grimminger et al., 2010). Because of its widespread involvement in numerous physiological and pathological processes (Thomas and Brugge, 1997; Dehm and Bonham, 2004; Lemmon and Schlessinger, 2010; Keller and Brummendorf, 2012), it is not surprising that the Src signaling pathway has been successfully targeted by multiple drug-discovery programs (Hopkins and Groom, 2002; Overington et al., 2006) and has been regarded as a promising new therapeutic approach for lung diseases (Richeldi et al., 2011; Oliveira et al., 2015; Cruz et al., 2016; da Silva et al., 2016) due to its anti-inflammatory and anti-fibrotic effects. In this line, nintedanib, an intracellular inhibitor that targets multiple tyrosine kinases, reduced lung-function decline and acute exacerbations in patients with idiopathic pulmonary fibrosis (Richeldi et al., 2011). Our group reported that dasatinib, a second-generation tyrosine kinase inhibitor, is regarded as a potential therapeutic strategy for the treatment of silicosis (Cruz et al., 2016).

Bosutinib is a third-generation oral multi-target inhibitor of tyrosine kinases (Remsing Rix et al., 2009), including the Src/Abl family, originally developed as a cancer treatment (Puttini et al., 2006; Vultur et al., 2008; Goldenberg, 2012; Amsberg and Koschmieder, 2013) and associated with fewer side effects than first- and second-generation inhibitors (Cortes et al., 2011; Khoury et al., 2012).

In this study, we hypothesized that bosutinib might reduce the inflammatory response, halt the progression of pulmonary fibrosis, and contribute to the improvement of lung function in a murine model of silicosis. For this purpose, we have investigated the mechanisms of action of bosutinib and their potential experimental efficacy in the treatment of silicosis.

## Materials and methods

The Ethics Committee of the Health Sciences Center, Federal University of Rio de Janeiro, approved this study (CEUA-CCS-012/14). All animals received humane care in compliance with the “Principles of Laboratory Animal Care” formulated by the National Society for Medical Research and the U.S. National Research Council “Guide for the Care and Use of Laboratory Animals,” and all efforts were made to minimize suffering.

### Animal preparation and experimental protocol

**Figure 1:**
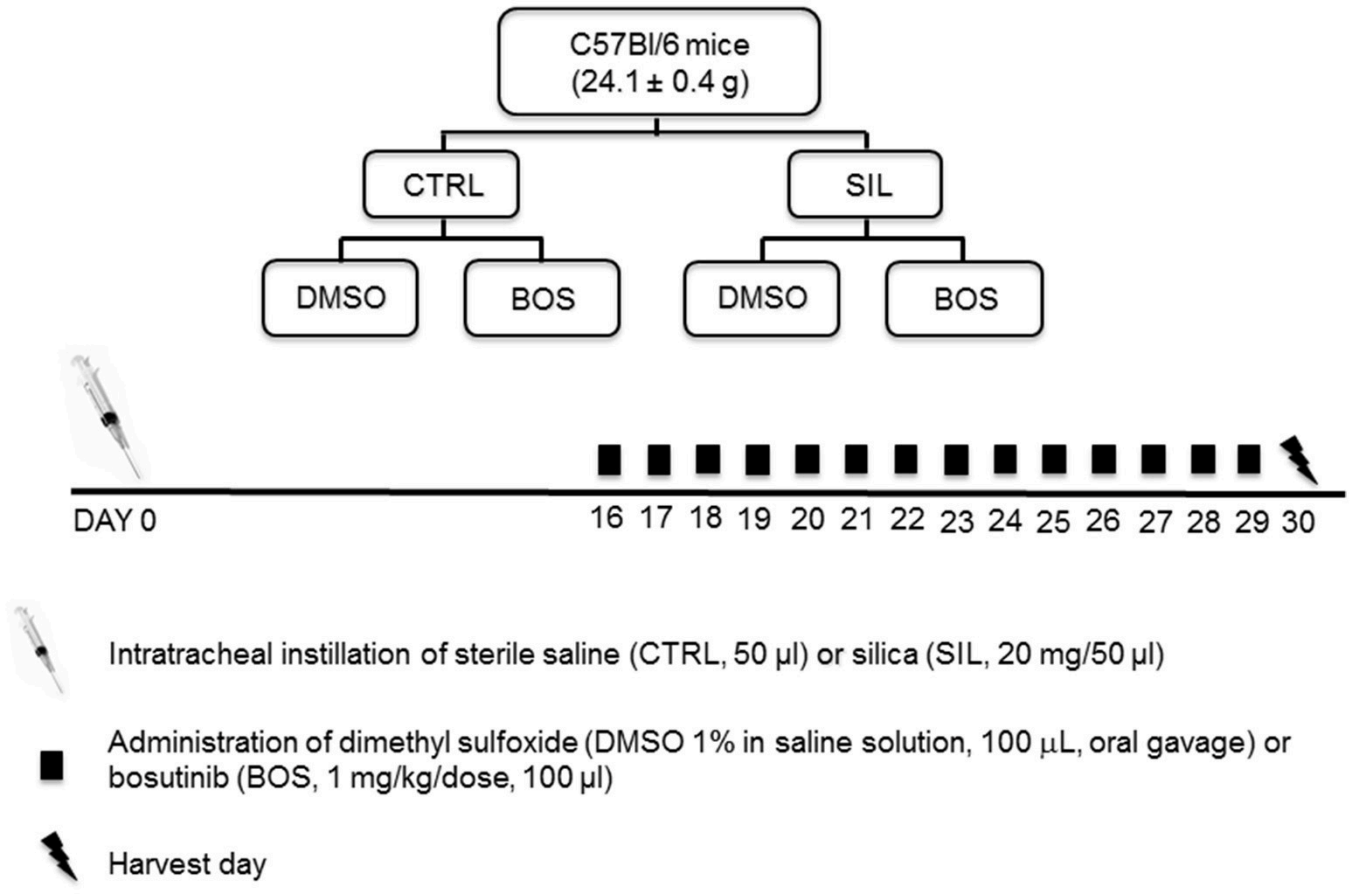
**Study design**. Sixty-four C57BL/6 female mice were divided into two groups: control (CTRL, *n* = 32) received sterile saline (50 μL) intratracheally (i.t.), while silicosis group (SIL, *n* = 32) received silica particles (20 mg in 50 μL saline, i.t.). Fifteen days after disease induction, the animals were randomized to receive dimethyl sulfoxide (DMSO 1% in saline solution, 100 μL, oral gavage) or bosutinib (BOS 1 mg/kg body weight in DMSO 1%, 100 μL, oral gavage).

A total of 64 C57BL/6 female mice (age 8–12 weeks, weight 24.1 ± 0.4 g) were assigned to two main groups: control (CTRL) and silicosis (SIL). Mice in the CTRL group received 50 μL sterile saline intratracheally (i.t.), while animals in the SIL group received a silica particle suspension (20 mg in 50 μL saline i.t.). Fifteen days after administration of saline or silica, animals were randomized to receive dimethyl sulfoxide (1% DMSO in saline solution, 100 μL, oral gavage; CTRL-DMSO and SIL-DMSO groups) or bosutinib (BOS 1 mg/kg body weight in 1% DMSO, 100 μL, oral gavage; CTRL-BOS and SIL-BOS groups), twice daily for 14 days (Figure 1). As bronchoalveolar lavage may affect lung morphological analysis and compromise lung function, 32 female C57BL/6 mice were used to evaluate lung mechanics and histology (*n* = 8/group), and another 32 female animals were used for analysis of total and differential cell counts in bronchoalveolar lavage fluid (BALF), thymus tissue, and lung-draining lymph nodes (*n* = 8/group). All mice had their clinical score evaluated at day at the harvest day (Araujo et al., 2010). The score consisted of analyzing the following parameters: presence of piloerection, altered respiration rate, fecal alteration, lacrimation/eyelid changes, contraction of the abdomen, lack of strength when grasping, change in body temperature, alert response (scape after touch), exploration of the environment and compromised locomotor activity. For every parameter present, we gave 1 point, and in the absence of the parameter analyzed, no points were given. Then, the points were computed for each mouse.

### Lung mechanics

Twelve hours after the last dose of bosutinib, 32 animals were sedated with diazepam [(Cristália, Itapira, SP, Brazil), 1 mg intraperitoneally (i.p.)], anesthetized with thiopental sodium [(Cristália, Itapira, SP, Brazil) 20 mg/kg i.p.], tracheotomized, paralyzed with vecuronium bromide [(Cristália, Itapira, SP, Brazil) 0.005 mg/kg intravenously (i.v.)], and ventilated with a constant-flow ventilator (Samay VR15; Universidad de la Republica, Montevideo, Uruguay) set as follows: respiratory rate = 100 breaths/min, tidal volume (V_T_) = 0.2 mL, and fraction of inspired oxygen (FiO_2_) = 0.21. The anterior chest wall was surgically removed and a positive end-expiratory pressure (PEEP) of 2 cmH_2_O applied. Airflow and tracheal pressure (Ptr) were measured. In an open chest preparation, Ptr reflects transpulmonary pressure (P_L_). After a 10-min ventilation period, static lung elastance (*E*st,L) and lung resistive (Δ*P*1,L) and viscoelastic/inhomogeneous pressures (Δ*P*2,L) were measured using the end-inflation occlusion method (Bates et al., 1985). All data were analyzed using ANADAT software (RHT-InfoData, Inc., Montreal, Quebec, Canada).

### Histological analysis

Immediately after determination of lung mechanics, a laparotomy was performed and heparin (Cristália, Itapira, SP, Brazil) 1,000 IU was injected into the vena cava. The trachea was clamped at end-expiration (PEEP = 2 cmH_2_O), and the vena cava and abdominal aorta were sectioned, leading to a massive hemorrhage and euthanasia by terminal bleeding. The left lung was then removed, flash-frozen by immersion in liquid nitrogen, fixed with Carnoy's solution and paraffin-embedded. Sections (4 μm thick) were cut and stained with hematoxylin and eosin. Lung morphometry analysis was performed using an integrating eyepiece with a coherent system consisting of a grid with 100 points and 50 lines of known length coupled to a conventional light microscope (Olympus BX51, Olympus Latin America-Inc., São Paulo, Brazil). The fraction area of lung occupied by collapsed or normal alveoli was determined by the point-counting technique (Weibel, 1990) across 10 random, non-coincident microscopic fields at 200× magnification. Briefly, points falling on collapsed or normal pulmonary areas were counted and divided by the total number of points in each microscopic field (Weibel, 1990). Additionally, the fraction area of granuloma was determined in 20 random non-coincident microscopic fields, at 400× magnification (Maron-Gutierrez et al., 2011; Lopes-Pacheco et al., 2013). Total cells and mononuclear and polymorphonuclear cell subpopulations in the alveolar septa and granuloma tissue were evaluated at 1,000× magnification in 10 random microscopic fields. Collagen fibers (Picrosirius polarization method) were computed in the alveolar septa and granuloma at 400× magnification using Image-Pro Plus 6.3 software (Media Cybernetics, Silver Spring, MD, USA; Maron-Gutierrez et al., 2011; Chen et al., 2013). Bronchi and blood vessels were carefully avoided during the measurements. The area occupied by fibers was determined by digital densitometric recognition. The results were expressed as the fraction area occupied by collagen fibers in the alveolar septa or in granuloma. An expert in lung pathology blinded to the experimental protocol performed all histological analyses.

### Immunohistochemistry for macrophages

Silanized slides containing 3-μm-thick tissue sections were deparaffinized in hot xylol (60–65°C) in an incubator for 10 min, then washed three times in cold xylol. For rehydration, slides were washed down through a graded ethanol series (100, 95, and 70%) at 70°C, rinsed successively in tap water and deionized water, and left in phosphate buffer (pH 7.4). Samples were then exposed to citric acid solution (10 mM pH 6.0) at high temperatures in a pressure cooker, for 1 min, for antigenic site recovery. Endogenous peroxidase was blocked by hydrogen peroxide 10 v/v (3%) and washed with phosphate-buffered saline (PBS). Unspecific proteins were blocked by slides immersion in 5% casein in phosphate buffer (pH 7.4) for 5 min. The slides were incubated with primary anti-(NOS)-2 antibody (1:100, Biorbyt Ltd., Cambridge, UK) for M1 macrophages and anti-CD163 antibody (1:100, Biorbyt Ltd., Cambridge, UK) for M2 macrophages, for 24 h. Slides were then incubated with a secondary antibody (Easylink One polymer HRP, EasyPath, São Paulo, Brazil) and diaminobenzidine (DAB) as a chromogen (Sigma-Aldrich). Subsequently, slides were counter-stained with Harris hematoxylin.

### Quantification of mediators in lung homogenates

Levels of interleukin (IL)-1β, tumor necrosis factor (TNF)-α, interferon (IFN)-γ, IL-4, transforming growth factor (TGF)-β, and vascular endothelial growth factor (VEGF) were quantified by ELISA in the lung homogenate. Lung tissue was homogenized in lysis buffer [PBS 1x, triton X 0.01%, 1 × Roche protease inhibitor cocktail (Roche Diagnostic, Mannheim, Germany)] using a glass Potter homogenizer with Teflon piston. The total amount of cytokines was quantified according to the manufacturer's protocol (Duo Set, R&D Systems, Minneapolis, MN, USA) and normalized to the total content of protein quantified by Bradford's reagent (Sigma-Aldrich, St Louis, MO, USA).

### Total and differential cell counts in bronchoalveolar lavage fluid, mediastinal lymph nodes, and thymus tissue

Thirty-two mice were euthanized for evaluation of total and differential cellularity in bronchoalveolar lavage fluid (BALF), mediastinal lymph nodes, and thymus. First, animals were euthanized with a lethal dose (100 mg/kg) of thiopental sodium (Cristália). The trachea was immediately cannulated, then flushed three times with 0.4 mL of 1% PBS solution in order to obtain BALF cells. BALF was then centrifuged (239 g, 10 min) and the cell pellet was resuspended in PBS. Cells from the lung-draining mediastinal lymph nodes and thymus were obtained through mechanical maceration of the organs. Total leukocytes were quantified with Türk solution in a Neubauer chamber.

T cells recovered from BALF, lymph nodes, and thymus were distinguished after staining with anti-mouse CD4 PE (eBioscience, SanDiego, CA, USA) for T helper cells, anti-mouse CD8 PECy5 (eBioscience, SanDiego, CA, USA) for T cytotoxic cells, and anti-mouse anti-CD4-FITC, anti-mouse anti-CD25-APC, and anti-mouse anti-Foxp3-PE (Treg kit, eBioscience, SanDiego, CA, USA) for T regulatory (Treg) cells. These cells were evaluated in a FACSCalibur flow cytometer (Becton Dickinson Biosciences, San Jose, CA, USA) and analyzed in FlowJo 7.6.5 software (TreeStarInc., Ashland, OR, USA).

### *In vitro* analysis of macrophages

RAW264.7 cells, a mouse peritoneal macrophage cell line, were obtained from American Type Culture Collection (Rockville, MD, USA) and maintained in culture using Dulbecco's Modified Eagle Medium (DMEM)—High Glucose supplemented with 10% fetal bovine serum, 1,000 U/mL penicillin/streptomycin, and 2 mM L-glutamine (Invitrogen, Life Technologies Grand Isle, NY, USA). Cells were plated in six-well plates (10^6^ cells/well) for 48 h. Fresh medium was then substituted, and cells were exposed to silica particles (100 μg per mL medium) for 24 h (21) or left incubated with regular medium. The supernatant was then removed, and cells were washed with 1X PBS and then incubated with bosutinib (100 ng/mL medium) or regular medium for 24 h. Once again, the supernatant was removed, cells washed with PBS, lifted using 2.5% Trypsin/EDTA (Invitrogen Life Technologies Grand Isle, NY, USA), and pelleted by centrifugation (600 g for 5 min).

A quantitative real-time reverse transcription polymerase chain reaction (PCR) was performed to measure mRNA expression of inducible nitric oxide synthase (iNOS), arginase-1, interleukin (IL)-10, IL-12, metalloproteinase (MMP)-9, tissue inhibitor of metalloproteinase (TIMP)-1, and caspase-3. Cells were lysed for RNA extraction through the RNeasy Plus Mini Kit (Qiagen, Valencia, CA, USA), following manufacturer recommendations. The total RNA concentration was measured by spectrophotometry in a Nanodrop ND-1000 system. First-strand cDNA was synthesized from total RNA using an M-MLV Reverse Transcriptase Kit (Invitrogen Life Technologies Grand Isle, NY, USA). Relative mRNA levels were measured with a SYBR Green detection system using ABI 7,500 real-time PCR (Applied Biosystems, Foster City, CA, USA). All samples were measured in triplicate. The relative level of each gene was calculated as the ratio of the study gene to the control gene (acidic ribosomal phosphoprotein P0 [36β4]) and given as the fold change relative to RAW cells incubated with regular medium. The following PCR primers were used: IL-10: forward 5′ TCC CTG GGT GAG AAG CTG-3′, reverse 5′ GCT CCA CTG CCT TGC TCT-3′; Arginase-1: forward 5′ GCT CAG GTG AAT CGG CCT TTT-3′, reverse 5′ TGG CTT GCG AGA CGT AGA C-3′; IL-12: forward 5′ AAC GCA GCA CTT CAG AAT CA-3′, reverse 5′ GAA GCA GGA TGC AGA GCT TC-3′; iNOS: forward 5′ CTTCAGGTATGCGGTATTGG-3′, reverse 5′ CAT GGT GAA CAC GTT CTT GG-3′; MMP-9: forward 5′ AGT CCG GCA GAC AAT CCT T-3′, reverse 5′ CCC TGT AAT GGG CTT CCT C-3′; TIMP-1: forward 5′ CAT GGA AAG CCT CTG TGG AT-3′, reverse 5′ GGG GAG ATG TGG ACT GTG A-3′; caspase-3: forward 5′ TAC CGG TGG AGG CTG ACT-3′, reverse 5′-GCT GCA AAG GGA CTG GAT-3′; 36β4: forward 5′ CAA CCC AGT TCT GGA GAA AC-3′, reverse 5′ GTT CTG AGC TCC CAC AGTGA-3′.

### Statistical analysis

Sample size calculation for testing the primary hypothesis (static lung elastance increased in the SIL group) was based on previous measurements (Lopes-Pacheco et al., 2013; Cruz et al., 2016) and on pilot studies. A sample size of 8 animals per group (considering one animal as dropout) was deemed to provide the appropriate power (1–β = 0.8) to identify significant (α = 0.05) differences in static lung elastance between SIL animals and those treated with BOS, with an effect size *d* = 1.9, a two-sided test, and a sample size ratio = 1 (G^*^Power 3.1.9.2, University of Düsseldorf, Germany). The normality of data (Kolmogorov–Smirnov test with Lilliefors' correction) and the homogeneity of variances (Levene median test) were tested. Parametric data are expressed as mean ± *SD*, and non-parametric data as median and interquartile range (IQR). To analyze all parameters associated with granuloma, the Mann–Whitney test for non-parametric data was used. Two-way ANOVA following Bonferroni's *post-hoc* test was used to compare all other parameters. *P*-values of 0.05 or less were considered significant. All tests were performed in the GraphPad Prism v6.0 statistical software package (GraphPad Software, La Jolla, CA, USA).

## Results

No animals died during experiments. Groups CTRL-DMSO and CTRL-BOS presented score 0 [0–0], SIL-DMSO presented a score of 0 [0–1], and SIL-BOS presented a score of 0 [0–1] with no statistical difference between groups. No statistical differences were observed between animas concerning their body weight during experiments. At day 0, animals weighted 24.1 ± 0.4 g. At day 15, CTRL animals weighted 24.9 ± 0.6 g, and SIL animals weighted 24.6 ± 0.7. At day 30, animals CTRL-DMSO weighted 26.3 ± 1.0 g, CTRL-BOS weighted 26.4 ± 0.8 g, SIL-DMSO weighted 25.5 ± 0.7 g, and SIL-BOS weighted 25.8 ± 0.5 g.

### Lung mechanics

**Figure 2:**
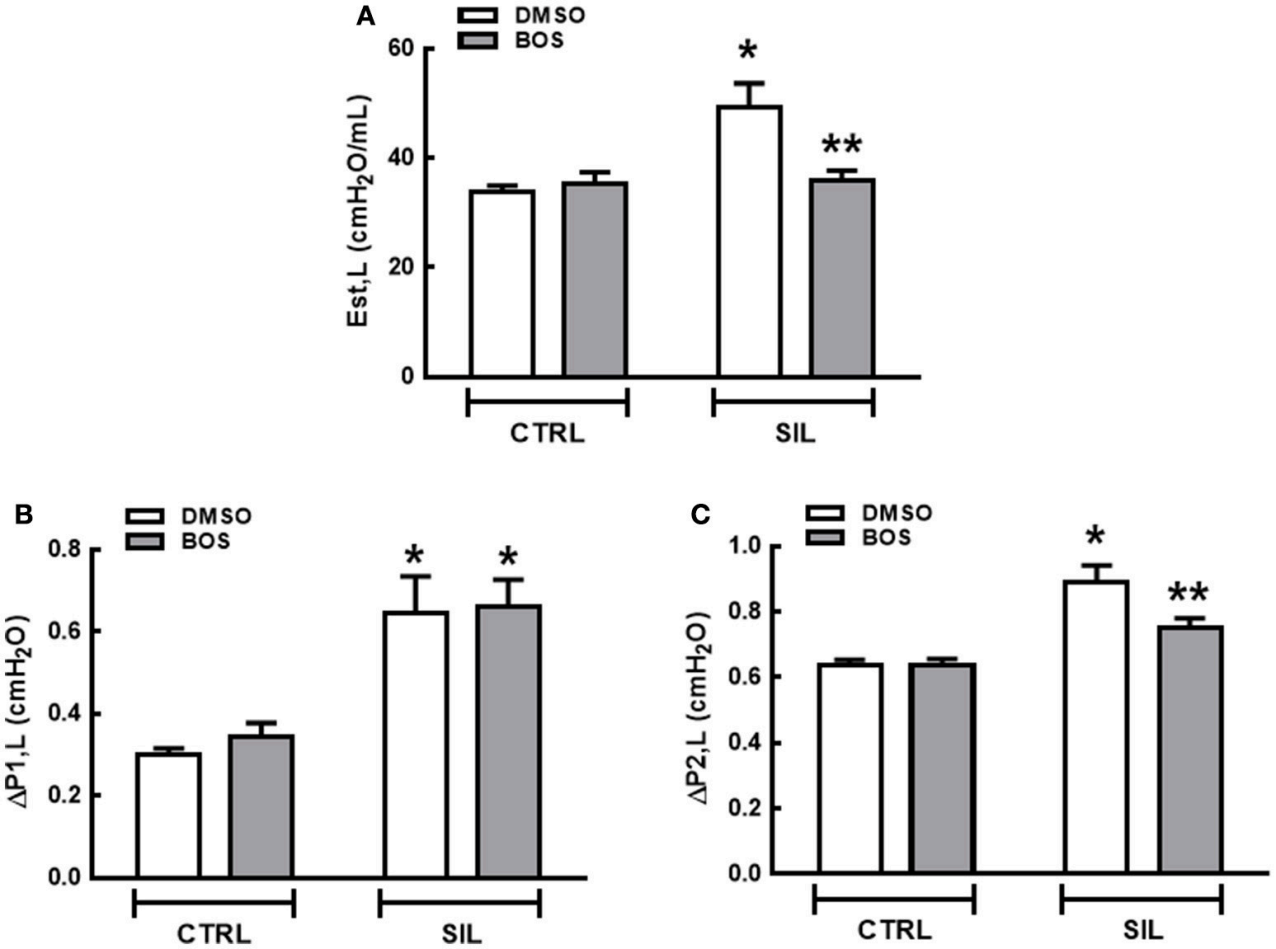
**Lung mechanics**. Static lung elastance (*Est,L*) **(A)**, resistive (ΔP1) **(B)**, and viscoelastic pressure (ΔP2) **(C)**. White bars, DMSO gray bars, BOS. Values are means + *SD* of eight animals per group. ^*^Significantly different from CTRL-DMSO group (*p* < 0.05). ^**^Significantly different from SIL-DMSO (*p* < 0.05).

Est,L (Figure 2A), ΔP1,L (Figure 2B), and ΔP2,L (Figure 2C) did not differ significantly between the CTRL-DMSO and CTRL-BOS groups. Est,L, ΔP1,L, and ΔP2,L were higher in SIL-DMSO compared to CTRL-DMSO animals (*p* = 0.0034, *p* = 0.0011, and *p* < 0.001, respectively). Bosutinib treatment reduced Est,L and ΔP2,L significantly in animals with silicosis (*p* = 0.0054 and *p* = 0.03, respectively); however, no significant differences were observed regarding ΔP1,L (*p* = 0.61; Figure 2).

### Lung morphometry and inflammation

**Figure 3:**
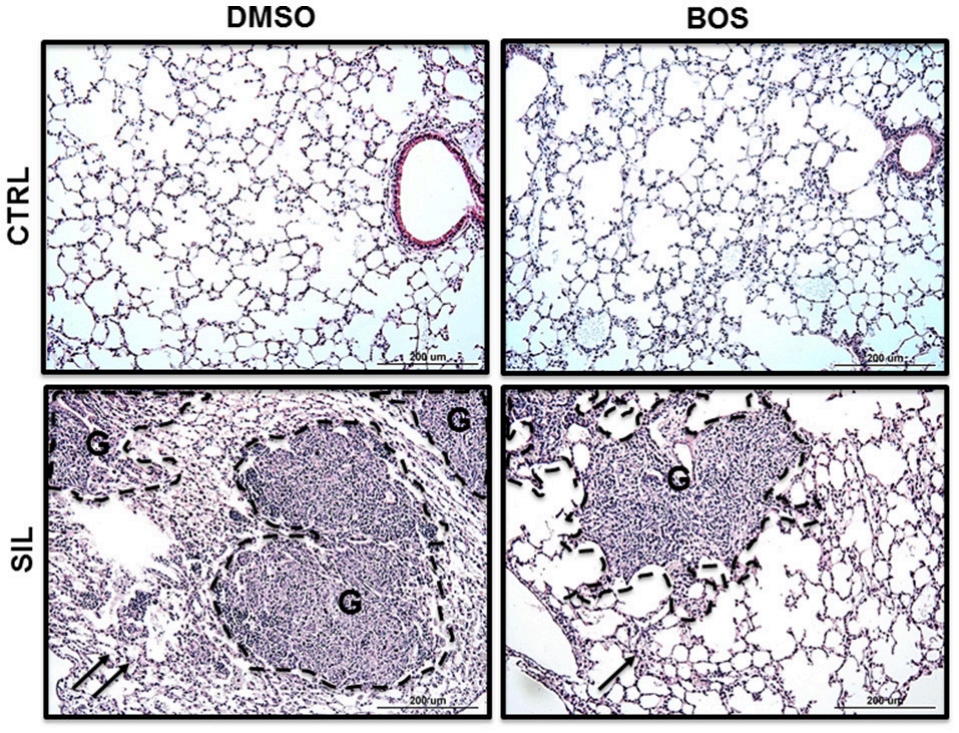
**Lung histology**. Representative photomicrographs (light microscopy) of lung parenchyma stained with hematoxylin and eosin from CTRL-DMSO, CTRL-BOS, SIL-DMSO, and SIL-BOS animals. G, granuloma. Arrows denote areas of alveolar collapse. Original magnification 200 ×. Scale bars = 200 μm.

Photomicrographs of lung parenchyma in both the CTRL-DMSO and CTRL-BOS groups showed preserved pulmonary structure (Figure 3). SIL-DMSO animals exhibited areas of granuloma with different sizes, associated with alveolar collapse, thickened alveolar septa with cell infiltration, and interstitial edema compared to CTRL-DMSO. After treatment with bosutinib, the fraction area of granuloma was reduced further in SIL-BOS compared to SIL-DMSO, and there was less alveolar collapse and interstitial edema (Figure 3).

SIL-DMSO was associated with a significant increase in the fraction area of alveolar collapse, fraction area of mononuclear cells, neutrophil count, and total cell count in the alveolar septa compared with CTRL-DMSO (*p* < 0.0001). Bosutinib treatment reduced the fraction area of alveolar collapse (*p* = 0.002), the fraction area of mononuclear cells (*p* = 0.0005), neutrophils (*p* = 0.0001), and total cells (*p* < 0.0001) in silicotic animals compared to SIL-DMSO (Table 1).

**Table 1:** **Lung morphometry**.

**Group**		**Normal (%)**	**Collapse (%)**	**Mononuclear cells (%)**	**Neutrophils (%)**	**Total cells (%)**
CTRL	DMSO	98.7 ± 0.8	1.3 ± 0.8	14.4 ± 0.8	2.8 ± 0.1	17.2 ± 0.8
	BOS	97.7 ± 1.6	2.3 ± 1.6	14.6 ± 2.2	2.8 ± 0.4	17.4 ± 2.2
SIL	DMSO	79.7 ± 6.4^*^	20.3 ± 6.4^*^	32.3 ± 3.5^*^	5.4 ± 0.8^*^	37.7 ± 3.7^*^
	BOS	89.6 ± 3.5^*^^,^^**^	10.4 ± 3.5^*^^,^^**^	27.3 ± 1.5^*^^,^^**^	3.9 ± 0.8^*^^,^^**^	31.2 ± 2.0^*^^,^^**^

*Fraction area of normal alveoli, collapsed alveoli, and lung hyperinflation. Values are mean ± SD of 10 animals per group. In the control (CTRL) group, mice received sterile saline (50 μL) intratracheally (i.t.), while in the silicosis group, animals received a silica particle suspension (20 mg in 50 μL saline, i.t.). Fifteen days after administration of saline or silica, animals were randomized to receive dimethyl sulfoxide (DMSO 1% in saline solution, 100 μL, oral gavage—CTRL-DMSO and SIL-DMSO groups) or bosutinib (BOS 1 mg/kg body weight in DMSO 1%, 100 μL, oral gavage—CTRL-BOS and SIL-BOS groups), twice a day for 14 days*.

* *Significantly different from CTRL-DMSO*.

** *Significantly different from SIL-DMSO (p < 0.05)*.

**Figure 4:**
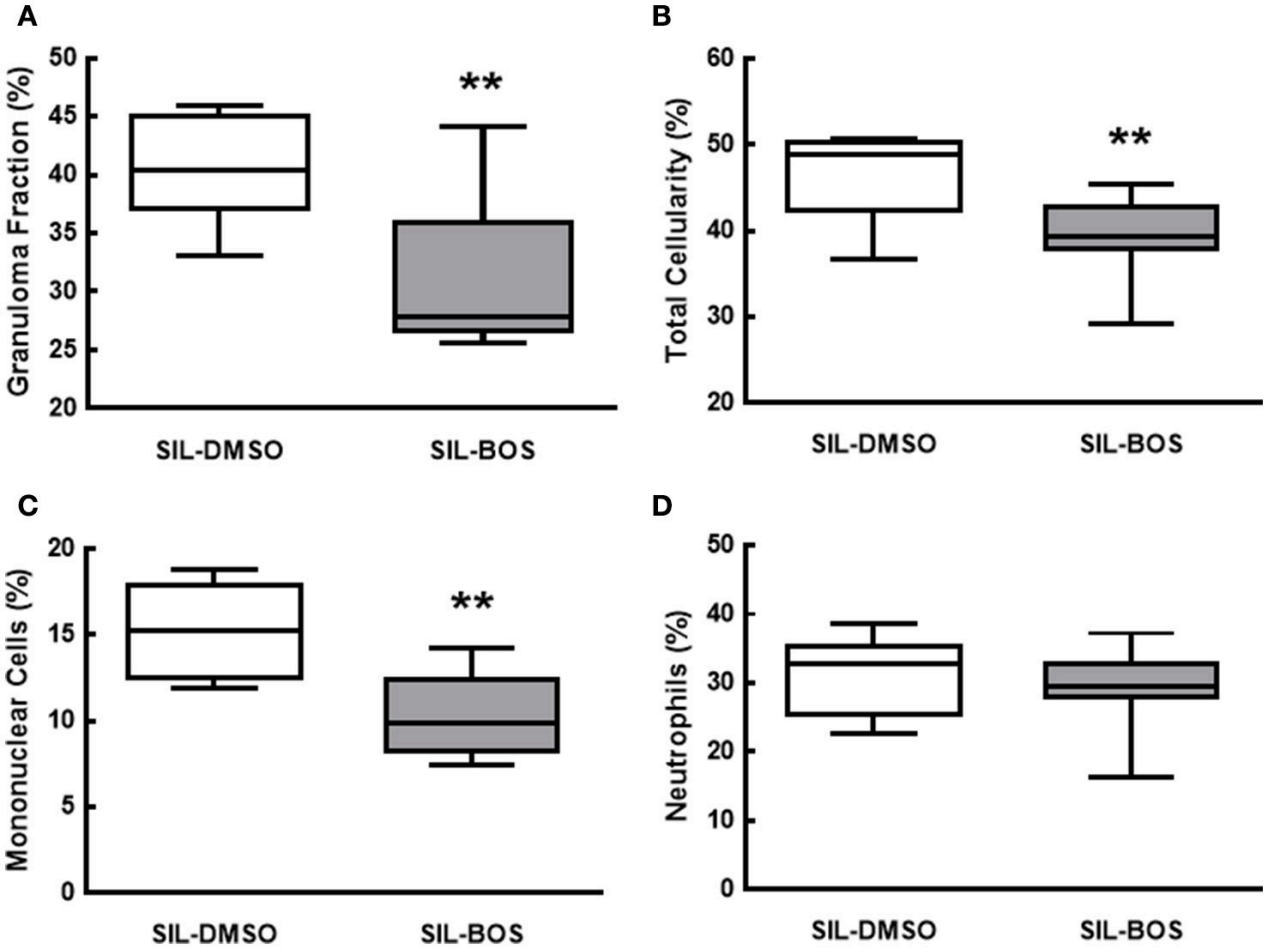
**Fraction area of granuloma in lung parenchyma (A)** and percent total cell count **(B)**, mononuclear cells **(C)**, and neutrophils **(D)**. All values were computed in 10 random, non-coincident fields of views per mouse. Boxes show the interquartile (25–75%) range, whiskers encompass the range (minimum–maximum), and horizontal lines represent median values of 8 animals per group. ^**^Significantly different from SIL-DMSO (*p* < 0.05).

Bosutinib therapy reduced the fraction area of granuloma (*p* = 0.01) (Figure 4A), as well as total cell counts (*p* = 0.03; Figure 4B) and mononuclear cell counts (*p* = 0.003) in granulomatous nodules (Figure 4C). No significant changes were observed in neutrophil counts within granuloma tissue (Figure 4D).

**Figure 5:**
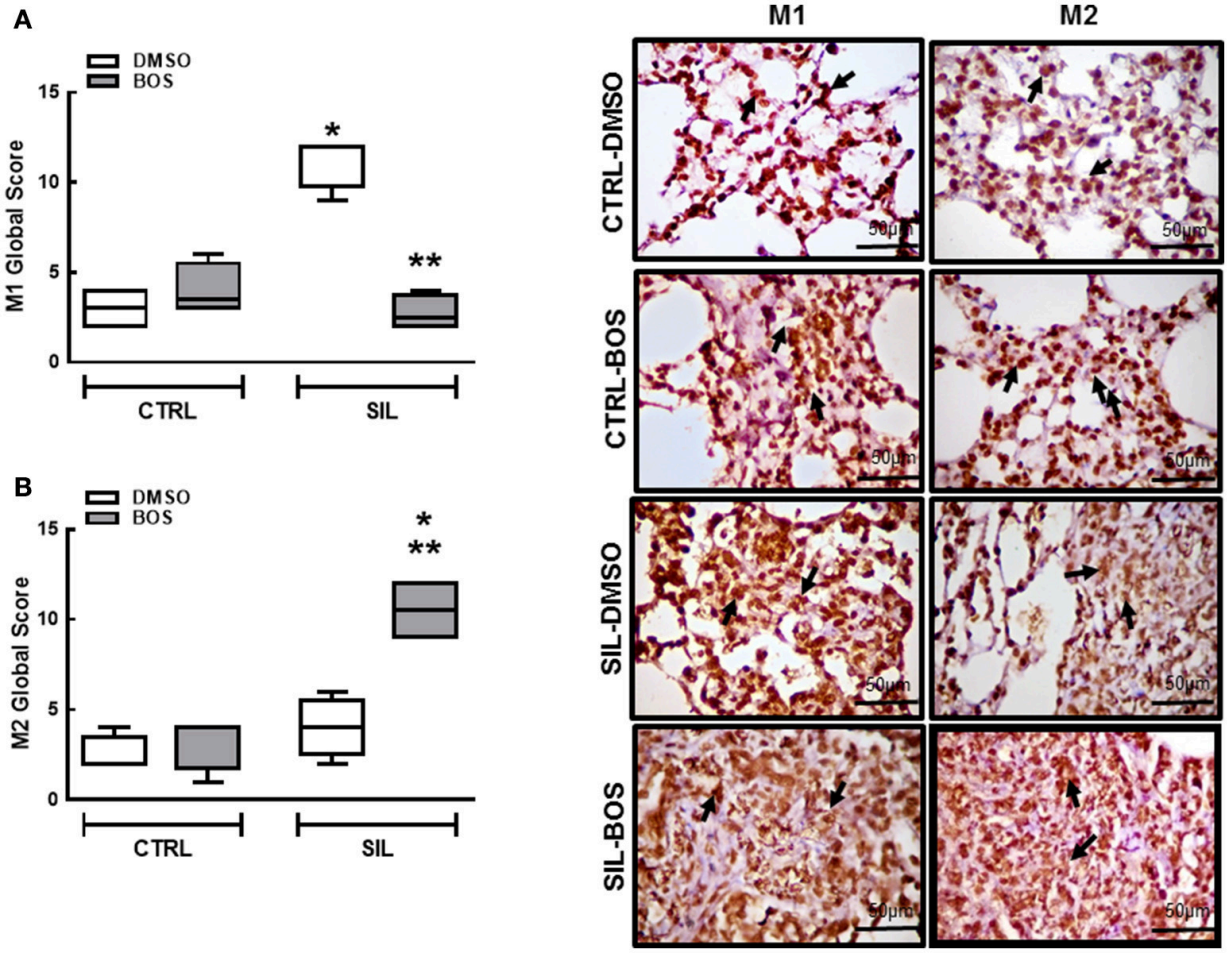
**Macrophage polarization: M1 and M2**. Representative immunohistochemistry photomicrographs of M1 (iNOS-positive) and M2 (arginase-positive) macrophages (right panels). Quantification of M1 **(A)** and M2 **(B)** (global score: severity × extension) in lung tissue of CTRL-DMSO, CTRL-BOS, SIL-DMSO, SIL-BOS. Arrows denote M1 or M2 cells. White bars, DMSO; gray bars, BOS. Boxes show the interquartile (25–75%) range, whiskers encompass the range (minimum–maximum), and horizontal lines represent median values of 8 animals per group. ^*^Significantly different from CTRL-DMSO group (*p* < 0.05). ^**^Significantly different from SIL-DMSO (*p* < 0.05).

The M1 (NOS2-positive) and M2 (CD168-positive) subpopulations were quantified in alveolar septa and granuloma for assessment of extension and severity (Figure 5). The product of these values was computed as the M1 or M2 global score. Bosutinib did not affect M1 or M2 global score in CTRL animals. M1 (pro-inflammatory) macrophages were increased in SIL-DMSO animals compared to CTRL-DMSO (*p* = 0.0286) and reduced in SIL-BOS compared to SIL-DMSO (*p* = 0.0265; Figure 5A). There were no significant changes in M2 (anti-inflammatory) macrophage subpopulations between CTRL-DMSO and SIL-DMSO groups (Figure 5B). However, there was a significant increase in M2 macrophages in SIL-BOS compared to CTRL-BOS (*p* = 0.025) and SIL-DMSO (*p* = 0.029) (Figure 5B).

**Figure 6:**
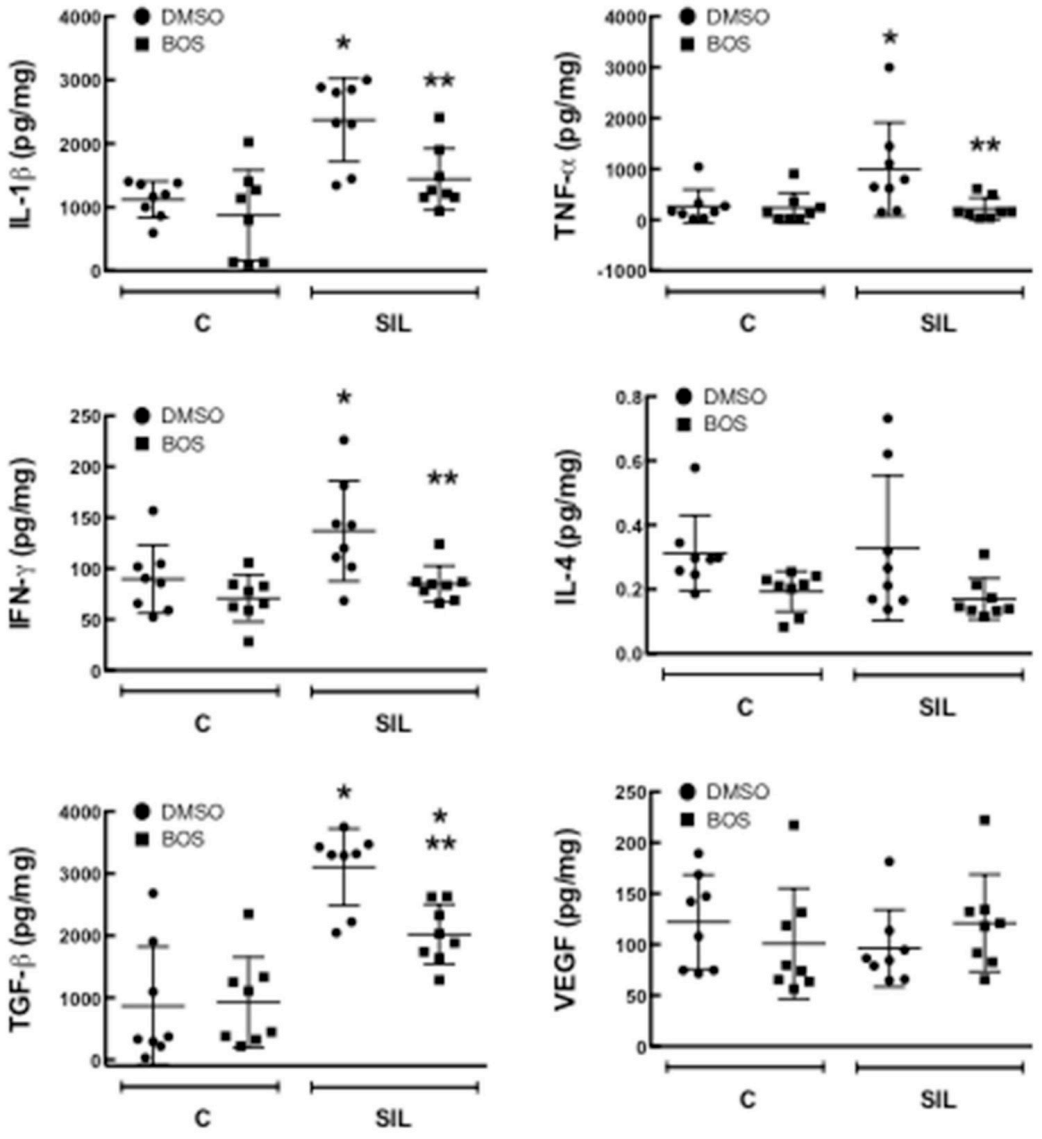
**Levels of cytokines and growth factors**. Quantification of protein levels of interleukin (IL)-1β, tumor necrosis factor (TNF)-α, interferon (IFN)-γ, IL-4, transforming growth factor (TGF)-β, and vascular endothelial growth factor (VEGF) quantified by ELISA in the lung homogenate. Black circle, DMSO; black square, BOS. Symbols represent individual animals. Lines represent median (interquartile range) of 8 animals per group. ^*^Significantly different from C-DMSO (*p* < 0.05). ^**^Significantly different from SIL-DMSO (*p* < 0.05).

Levels of IL-1β, TNF-α, IFN-γ, and TGF- β were increased in lung homogenates of SIL-DMSO compared to CTRL-DMSO (*p* = 0.0006; *p* = 0.0448; *p* = 0.0372; *p* < 0.0001, respectively). Treatment with bosutinib reduced levels of IL-1β, TNF-α, and IFN-γ (*p* = 0.0124, *p* = 0.0295, *p* = 0.0192, respectively) and minimized levels of TGF-β (*p* = 0.0258) compared to SIL-DMSO. No differences were observed in IL-4 and VEGF levels among groups (Figure 6).

### *In vitro* analysis

**Figure 7:**
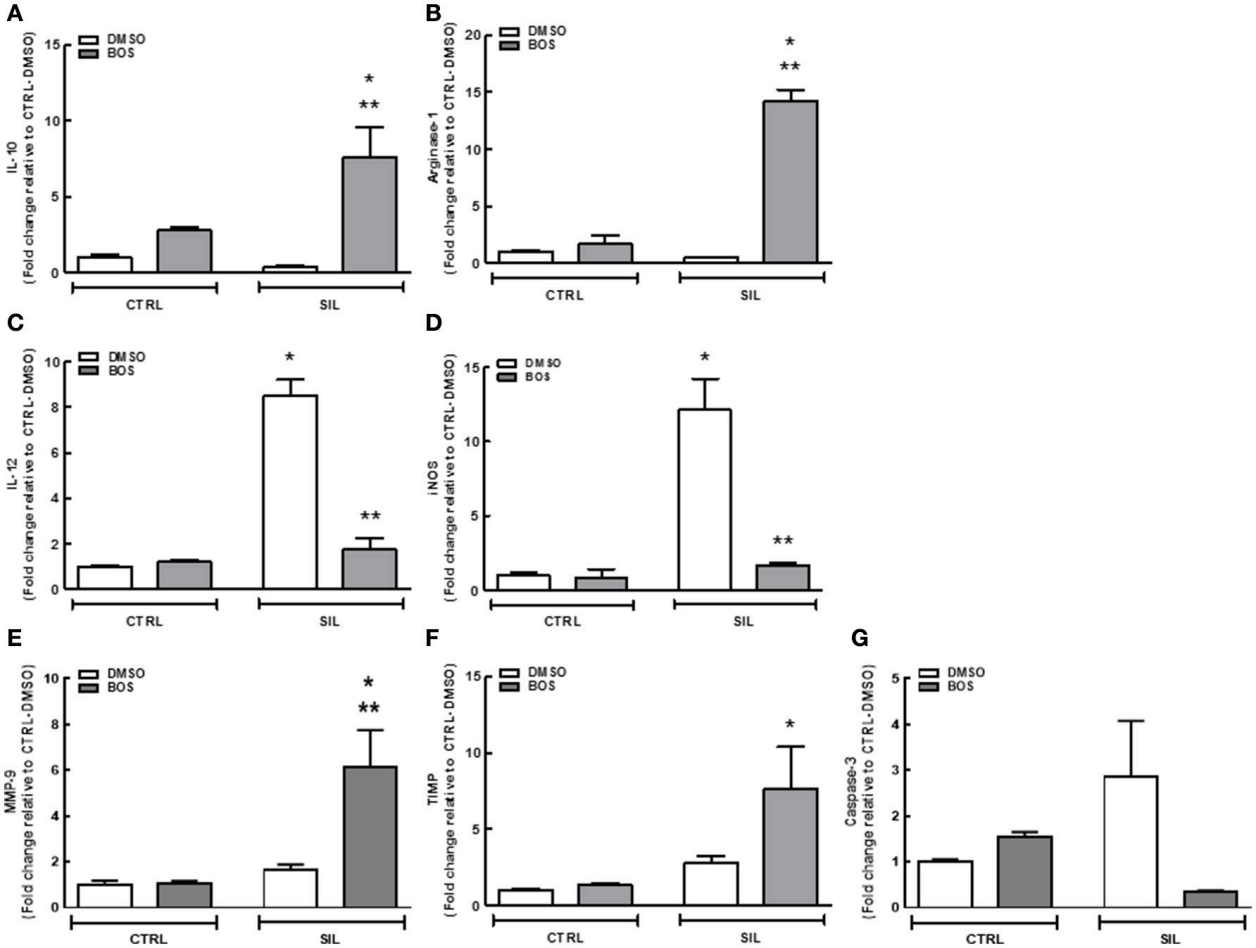
**Expression of interleukin (IL)-10 (A)**, arginase (Arg)-1 **(B)**, IL-12 **(C)**, inducible nitric oxide synthase (iNOS) **(D)**, metalloproteinase (MMP)-9 **(E)**, tissue inhibitor of metalloproteinase (TIMP) **(F)**, and caspase-3 **(G)** in macrophages exposed to saline or silica and treated with DMSO or bosutinib (BOS). White bars, DMSO; gray bars, BOS. Values are means + *SD* of 8 animals per group. ^*^Significantly different from C-DMSO (*p* < 0.05). ^**^Significantly different from SIL-DMSO (*p* < 0.05).

Macrophages exposed to silica and treated with bosutinib showed an increase in expression of IL-10, and anti-inflammatory cytokine (*p* = 0.0004; Figure 7A), and arginase-1, a classical marker of M2 macrophages (*p* = 0.0002; Figure 7B), compared to those exposed to silica and treated with DMSO; reduced expression of the pro-inflammatory cytokine IL-12 (*p* = 0.007; Figure 7C) and of iNOS, a marker of M1 macrophages (*p* = 0.019), was also observed (Figure 7D). In macrophages exposed to silica and treated with DMSO, IL-12 (*p* = 0.003; Figure 7C), and iNOS (*p* = 0.017) expressions (Figure 7D) were increased compared to macrophages not exposed to silica but treated with DMSO. In addition, there was increased expression of MMP-9 (*p* = 0.047; Figure 7E) and TIMP-1 (*p* = 0.026; Figure 7F) in macrophages exposed to silica and treated with bosutinib compared to those treated with DMSO. No significant differences among groups were observed regarding expression of the pro-apoptotic mediator caspase-3 (Figure 7G).

### Flow cytometry

**Figure 8:**
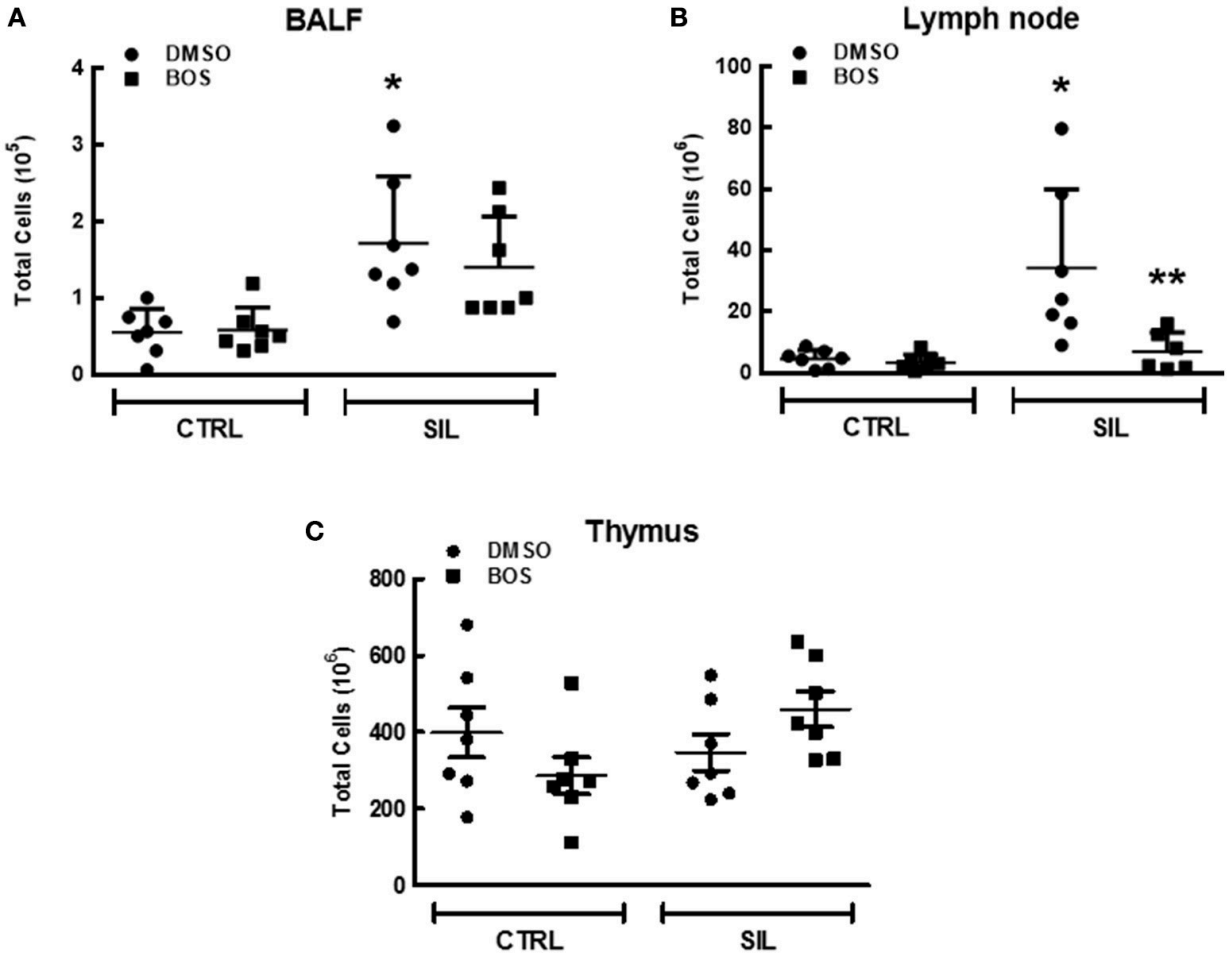
**Total inflammatory cell counts in bronchoalveolar lavage fluid (BALF) (A)**, lymph node **(B)**, and thymus tissue **(C)**. Black circle, DMSO; black square, BOS. Symbols represent individual animals. Lines represent median (interquartile range) of 6 or 8 animals per group. ^*^Significantly different from C-DMSO (*p* < 0.05). ^**^Significantly different from SIL-DMSO (*p* < 0.05).

Leukocyte counts were higher in the BALF (*p* = 0.02) and lung-draining lymph nodes (*p* = 0.015) of SIL-DMSO compared to CTRL-DMSO animals (Figures 8A,B). In SIL-BOS animals, leukocyte counts in the lung-draining lymph nodes were lower than those of SIL-DMSO animals (*p* = 0.005; Figure 8B). There was no difference in thymus cellularity across groups (Figure 8C).

**Figure 9:**
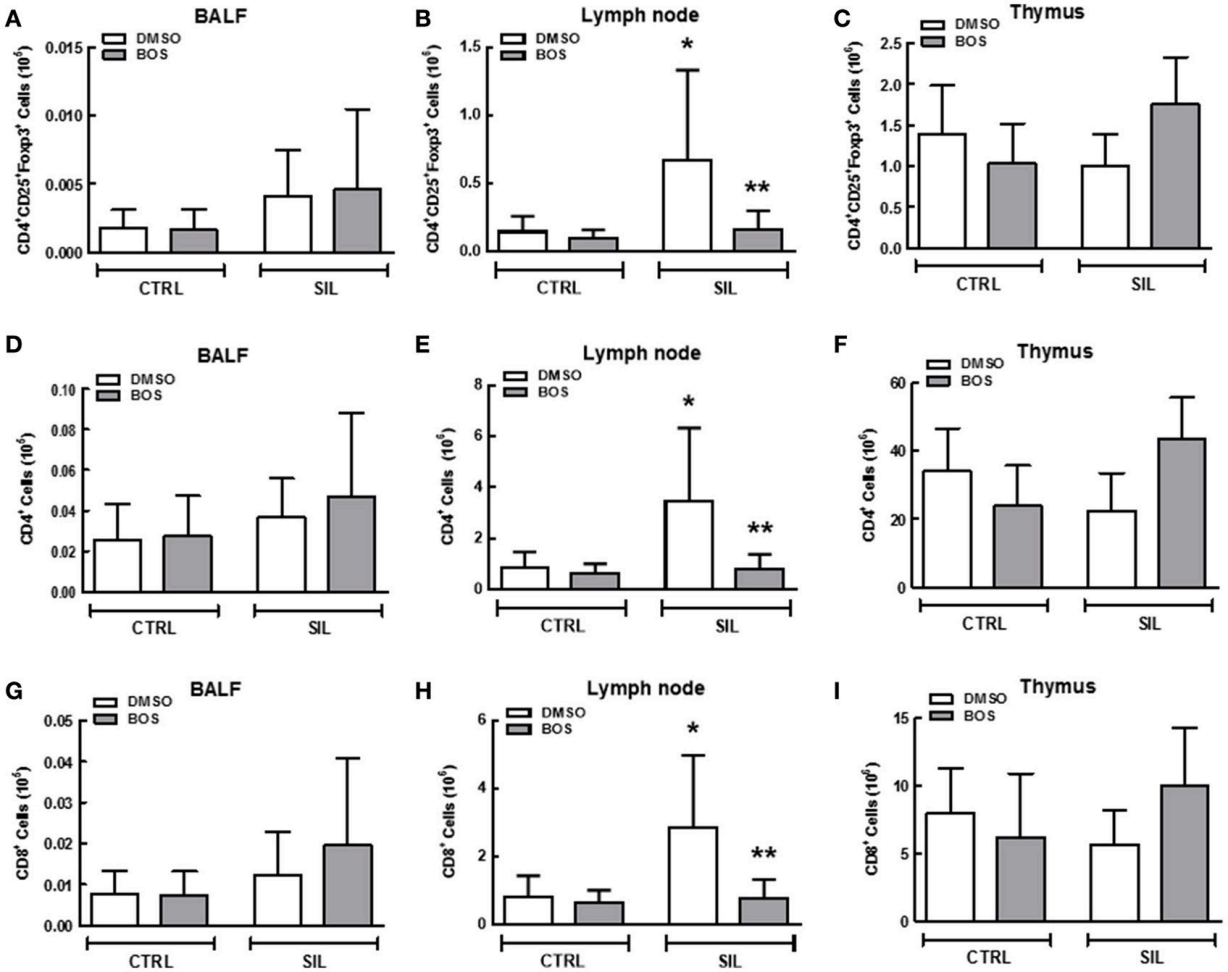
**Regulatory T cells, helper T cells, and killer T cells in bronchoalveolar lavage fluid (BALF), lymph nodes, and thymus tissue**. Quantification of total CD4^+^CD25^+^Foxp3^+^ cells (first vertical line), CD4^+^ cells (second vertical line), and CD8^+^ cells (third vertical line) in BALF **(A,D,G)**, lymph node **(B,E,H)**, and thymus tissue **(C,F,I)**, respectively, of CTRL-DMSO, CTRL-BOS, SIL-DMSO, and SIL-BOS animals. White bars, DMSO; gray bars, BOS. Values are means + *SD* of 8 animals per group. ^*^Significantly different from C-DMSO (*p* < 0.05). ^**^Significantly different from SIL-DMSO (*p* < 0.05).

We observed an increase in absolute counts of Treg cells (*p* = 0.030) (Figure 9B), CD4^+^ lymphocytes (*p* = 0.007; Figure 9E), and CD8^+^ lymphocytes (*p* = 0.026; Figure 9H) in the lung-draining lymph nodes of SIL-DMSO vs. CTRL-DMSO animals. There was a significant reduction in absolute number of Treg (*p* = 0.03; Figure 9B), CD4^+^ (*p* = 0.014; Figure 9E), and CD8^+^ cells (*p* = 0.016; Figure 9H) in SIL-BOS compared to SIL-DMSO animals. No significant differences in the number of these cells in BALF or thymus were observed between groups (Figures 9A,C,D,F,G,I).

### Lung fibrosis

**Figure 10:**
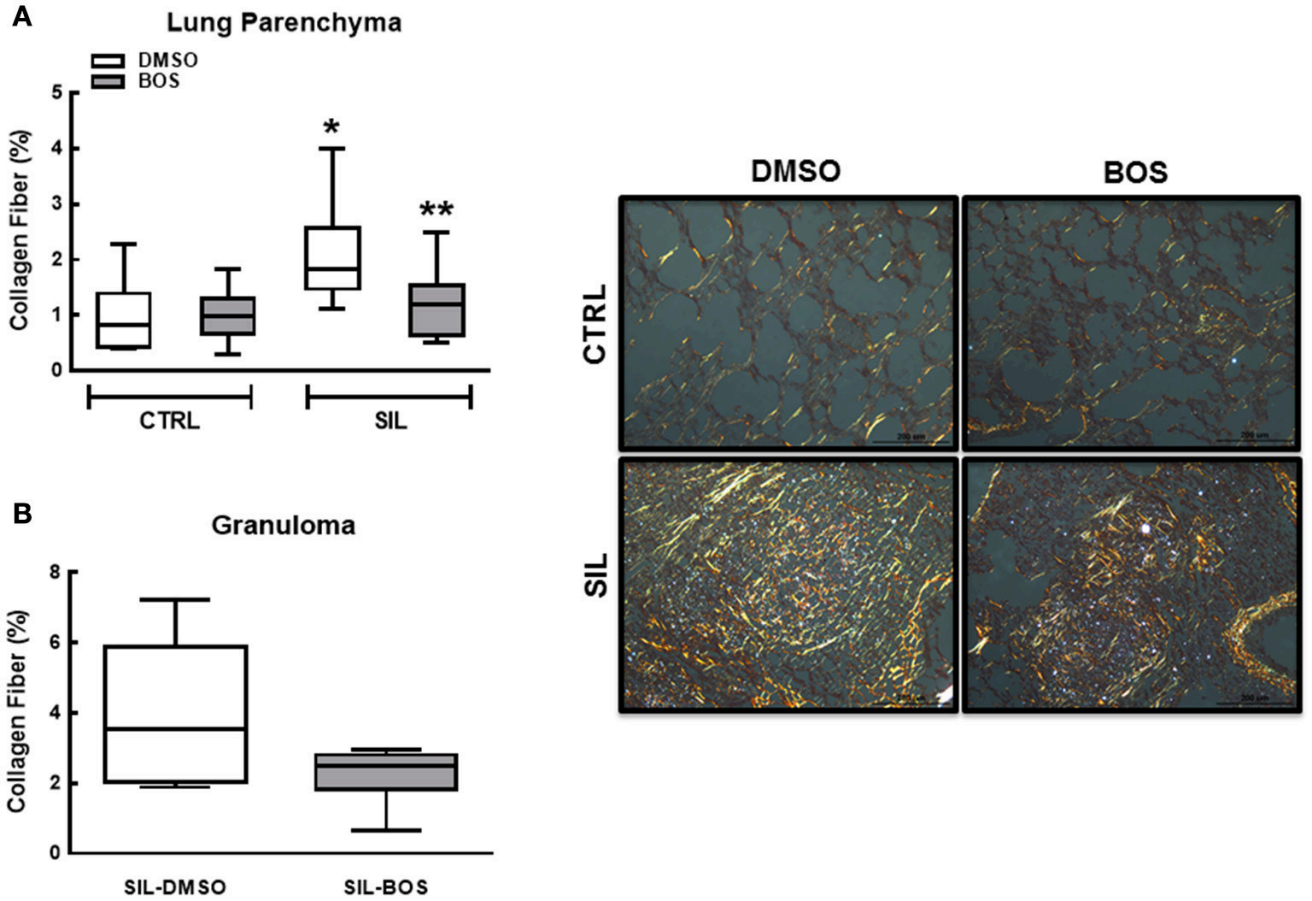
**Collagen fibers**. Representative photomicrographs (polarized light microscopy) of lung parenchyma and granuloma tissue stained with Picrosirius Red (right panels). Collagen fibers are shown in orange. Original magnification 200 ×. Scale bars = 200 μm. Left panels depict collagen fiber content quantification in lung parenchyma (upper panel, **A**) and granuloma (lower panel, **B**). White bar, DMSO; gray bar, BOS. Boxes show the interquartile (25–75%) range, whiskers encompass the range (minimum–maximum), and horizontal lines represent median values of 8 animals per group. ^*^Significantly different from C-DMSO (*p* < 0.05). ^**^Significantly different from SIL-DMSO (*p* < 0.05).

The percentage of collagen fibers quantified in lung parenchyma was increased in SIL-DMSO compared to CTRL-DMSO animals (*p* = 0.02; Figure 10A). In silicotic animals, treatment with bosutinib reduced the percentage of collagen fibers as compared to DMSO treatment alone (*p* = 0.04; Figure 10A). The amount of collagen fibers within granuloma tissue did not differ between the SIL-DMSO and SIL-BOS groups (*p* = 0.123; Figure 10B).

## Discussion

Under the conditions of this study, bosutinib therapy of silicotic animals had a variety of positive effects, including reduction in: (1) fraction area of collapsed alveoli and granuloma, as well as in neutrophil and macrophage counts in lung tissue; (2) M1 macrophages in alveolar septa and granuloma, (3) IL-1β, TNF-α, IFN-γ, and TGF-β levels in lung homogenates, (4) total cellularity, Treg, CD4^+^, and CD8^+^ populations in lung-draining lymph nodes, and (5) collagen fiber content in lung parenchyma. Additionally, lung mechanics improved and M2 macrophages increased in alveolar septa and granuloma. Our *in vitro* experiment showed that bosutinib reduced expression of iNOS and IL-12 and increased expression of IL-10, arginase, MMP-9, and TIMP-1 in macrophages exposed to silica. To the best of our knowledge, this was the first study to analyze the potential therapeutic effects of bosutinib on lung function, inflammation, and remodeling in experimental silicosis.

In the murine model used in the present study, silicosis was induced by a single exposure to crystalline silica, which, after 15 days, led to lung mechanical and histological changes that resembled those of human silicosis (Faffe et al., 2001; Maron-Gutierrez et al., 2011; Lopes-Pacheco et al., 2013). In addition, we have decided to use a C57BL/6 mouse strain, which is a prototypical Th1-type mouse strain. Additionally, when compared to other mouse strains, C57BL/6 macrophages present higher levels of TNF-α and IFN-γ and, higher phagocytic activity when stimulated (Watanabe et al., 2004).

In our study, CTRL animals were treated with DMSO. Even though DMSO may result in lung inflammation, pilot studies compared saline with DMSO and found no significant differences in lung mechanics and morphometry or collagen fiber content between groups (Oliveira et al., 2015; Cruz et al., 2016; da Silva et al., 2016). Mice exposed to silica exhibited higher Est,L and ΔP2,L values, which were associated with the presence of silicotic granulomas, alveolar collapse, thickening of alveolar septa, infiltration of inflammatory cells in lung parenchyma, and collagen fiber deposition. The increase in airway resistive pressure (ΔP1,L) may have been due to the greater number of cells in the bronchi associated with areas of lumen obstruction (Faffe et al., 2001; Borges et al., 2002; Maron-Gutierrez et al., 2011; Lopes-Pacheco et al., 2013; Cruz et al., 2016). Bosutinib decreased both Est,L and ΔP2,L likely due to reduction in alveolar collapse, interstitial edema, thickening of alveolar septa, cell infiltration and collagen fiber content, whereas Δ*P*1,L did not change after therapy which may be attributed to the presence of the silica particle adhere to the airway and airway hyperreactivity (Ferreira et al., 2013).

Silicosis is classically characterized by mononuclear cell aggregation with mineral particles and fibrosis. Macrophages are the key innate immune system cells implicated in this response, and orchestrate the entire pathophysiology of silicosis. Briefly, silica particles are phagocytized by macrophages that are activated, thus inducing oxidative stress, mitochondrial injury, lysosome injury, and apoptosis (Setoguchi et al., 1996; Fubini and Hubbard, 2003). Silica particles induce the release of metalloproteases, free radicals, and pro-inflammatory mediators, such as: IL-1β and TNF-α through activation of the nuclear factor (NF)-κB pathway (Rimal et al., 2005; Greenberg et al., 2007), which is involved in the recruitment of more inflammatory cells to the site of the lesion—including neutrophils, which are involved in the lung fibrosis process (Maron-Gutierrez et al., 2011).

Furthermore, lymphocytes (predominantly CD4^+^ T cells, but also numerous CD8^+^ T cells and natural killer cells) are known to be abundant in the lesions of silicosis (Davis et al., 2001). In our study, silica-exposed mice exhibited accumulation of lymphocytes in alveolar spaces, lung parenchyma, granuloma, enlarged bronchial-associated lymphoid tissues, and thoracic lymph nodes, which is consistent with this feature. Previous studies showed that Th1 and Th2 cytokines are involved in silicosis, but Th1/Th2 polarization during the development of silicosis is still a matter of debate. Several evidences suggest each plays a distinct role. Specifically, Th1 response is inflammation is upregulated in the acute phase while Th2 cytokines have only been associated with fibrosis in the later stages (Garn et al., 2000). It might explain the increased levels of IFN-γ in lung homogenates in SIL-DMSO animals. In addition, Treg cells play a crucial role in modulation of immune homeostasis by regulating Th1/Th2 polarization, but their possible implication in silicosis has been explored only in the past few years. Liu et al. suggested that depletion of Tregs attenuated the progression of silica-induced lung fibrosis, reduced Th1 response, and shifted the Th1/Th2 balance toward a Th2 phenotype (Liu et al., 2014). In our sample, bosutinib was able to reduce the number of Treg cells and IFN-γ levels which might be associated with less collagen deposition in lung parenchyma.

Several tyrosine kinase pathways have been found to play essential roles in the pathophysiology of silicosis. At therapeutic dosages, bosutinib, an ATP-competitive tyrosine kinase inhibitor, inhibits the activity of Bcr-Abl and Src-family kinases including Src, Yes, Fgr, Fyn, Lck, Lyn, Hck, and Blk. Bosutinib was first approved to treat chronic myeloid leukemia in patients resistant or intolerant to prior lines of therapy (Thomas and Brugge, 1997; Goldenberg, 2012). Besides its effect on malignant cells, bosutinib also blocks several inflammatory cell pathways, since tyrosine kinases are closely involved in a wide variety of signaling pathways of the hematopoietic system, including those mediated by receptors of specific antigens, integrin, cytokines, and toll-like mediated receptors (Page et al., 2009). Thus, bosutinib inhibits lymphocyte activation and proliferation and suppresses neutrophil and macrophage activation and chemotaxis (Zarbock and Ley, 2011; Futosi et al., 2012), due to its inhibition of Lck (predominantly expressed in T cells), Blk (in B cells), Lyn and Fgr (in myeloid cells), and Src, Yes, and Fyn (in leukocytes in general; Thomas and Brugge, 1997). The dose of bosutinib chosen for the present study (1 mg/kg) was based on pilot studies conducted in our lab. The reduction of macrophage and neutrophil counts in lung parenchyma and lymphocytes in lung-draining lymph nodes effected by bosutinib in this study might be explained by the ability of this compound to disrupt leukocyte adhesion, activation, and migration (Zarbock and Ley, 2011).

Oxidative stress, generated either directly by silica particles or indirectly by inflammatory cells, triggers activation of tyrosine kinases that are involved in the dimerization and nuclear translocation of NF-κB (Kang et al., 2006), which regulates genes that control physiological processes including innate immune response and inflammation (Di Giuseppe et al., 2009) and that promote macrophage polarization toward the M1 phenotype (Portaa et al., 2009). Bosutinib was able to reduce M1 macrophage and increase M2 macrophage counts in lung parenchyma and granuloma tissue from silicotic mice in our sample. This suggests that bosutinib might inhibit this signaling pathway, since NF-κB activation in macrophages is Src-dependent (Kang et al., 2006). M2 macrophages can resolve inflammation through high endocytic clearance abilities; production of autocrine and paracrine trophic factors; reduced pro-inflammatory cytokine secretion (Wang et al., 2007); increased expression of arginase-1, which inhibits the production of pro-inflammatory and oxidative nitric oxide (Mosser, 2003); and enhanced expression of the IL-1 receptor antagonist (which inhibits the effects of pro-inflammatory IL-1), the mannose receptor, and chitinase-like 3 (Wilson et al., 2004).

The role of M2 macrophages in fibrosis is controversial. They have been associated with fibrosis development, since their numbers are increased in pulmonary fibrosis (Endo et al., 2003; Boorsma et al., 2013). On the other hand, new evidence suggests they may not be the cause but a consequence of fibrosis, more specifically of a failed attempt to clear excess extracellular matrix and thus trigger fibrosis resolution. In liver fibrosis models, M2 macrophages were not required for development of fibrosis (Herbert et al., 2004), but were shown to be important for its resolution (Pesce et al., 2009; Weng et al., 2014). Furthermore, secretion of TIMP-1 is involved in spontaneous resolution of liver fibrosis by the combination of a net reduction and suppression of apoptosis; conversely, downregulation of TIMP-1 has been found to exacerbate liver injury and fibrosis (Yoshiji et al., 2002). M2 macrophages have been shown to produce MMP-9, which may contribute toward attenuation of fibrotic lesions (Bourlier et al., 2008). In the context of lung fibrosis, Misson et al. addressed the question of whether M2 macrophages were associated with collagen fiber deposition in the lungs in a murine model of single intratracheal instillation of silica particles. The authors observed that the upregulation of M2-associated genes such as arginase-1 mRNA was not associated with the severity of lung fibrosis (Misson et al., 2004). Moreover, M2 macrophages express different mannose receptors, also considered classical M2 markers, which appear to be responsible for the uptake of extracellular matrix components (Weng et al., 2014). In a previous study by our group, we showed that dasatinib, another tyrosine kinase inhibitor, increased M2 macrophage counts and reduced collagen deposition in lung parenchyma and granuloma (Cruz et al., 2016). Our results showed that the antifibrotic effects of bosutinib might be explained by its anti-inflammatory properties, by the antifibrotic effects of M2 macrophages, downregulation of Treg cells, reduced levels of the pro-fibrotic mediator TGF-β and by direct pharmacological inhibition of PTK, which is associated with profibrotic receptors such as FAK (Reiske et al., 2000; Hauck et al., 2002; Bourlier et al., 2008; Siesser et al., 2008; Lagares et al., 2012; Zhao et al., 2016).

In the present study, bosutinib reduced inflammation and induced several hallmark features of regulatory-like macrophages, subsequently decreasing lung fibrosis, in mice with experimental silicosis. Whether other tyrosine kinase inhibitors might exert similar effects remains unclear, as does their clinical potential for this indication. As noted above, in a previous study by our group (Cruz et al., 2016), the second-generation tyrosine kinase inhibitor dasatinib was shown to be partially effective at improving lung mechanics and reducing remodeling in a murine model of silicosis. Clinically, dasatinib has been associated with adverse events such as diarrhea, pleural effusion, and dyspnea in patients with breast cancer and melanoma (Finn et al., 2011; Kluger et al., 2011). Nintedanib (formerly known as BIBF 1120) is an intracellular inhibitor that targets multiple tyrosine kinases. In a phase II trial patients with idiopathic pulmonary fibrosis, nintedanib at a dose of 150 mg twice daily reduced lung-function decline and acute exacerbations, but several patients discontinued treatment due to adverse effects (Richeldi et al., 2011).

This study presents limitations: (1) bosutinib was administered 15 days after silica administration, when the disease was already established, but further studies are required evaluating the effects of bosutinib late in the course of disease; and (2) we did not evaluate the impact of bosutinib on different components of extracellular matrix (ECM), as well as MMP and TIMPS in the lung, but focused on the main component of ECM (collagen fiber) modified in silicosis (Hubbard et al., 2005).

## Conclusions

In a manner consistent with its Src-family tyrosine-kinase inhibition profile, bosutinib reduced inflammation in the lung and lung-draining lymph nodes and mitigated fibrosis in the lung parenchyma of mice subjected to induction of experimental silicosis. Although, the precise mechanism behind this effect remains unknown, this inhibitor may represent an interesting alternative for treatment of fibrotic diseases.

## Author contributions

PC, PR, and FC conceived and designed the experiments; PC, AC, GP, JS, JK, PO, VC, and FC performed the experiments and analyzed the data. PC, AC, FC, PR, PO, and JK coordinated data collection and data quality assurance. PC, FC, and PR wrote the first draft of the manuscript. All authors participated in the manuscript writing process and read and approved the final version.

## Funding

This study was supported by Conselho Nacional de Desenvolvimento Científico e Tecnológico/Ministério da Saúde/DECIT [469716/2014-2, 465064/2014-0, and 400462/2014-1 to PR], Fundação Carlos Chagas Filho de Amparo à Pesquisa do Estado do Rio de Janeiro [E-26/103.118/2 to PR], and the European Community's Seventh Framework Programme under grant agreement no. HEALTH-F4-2011-282095 (TARKINAID project).

### Conflict of interest statement

The authors declare that the research was conducted in the absence of any commercial or financial relationships that could be construed as a potential conflict of interest.

## References

[B1] Ahluwalia M. S. de Groot J. Liu W. M. Gladson C. L. (2010). Targeting SRC in glioblastoma tumors and brain metastases: rationale and preclinical studies. Cancer Lett. 298, 139–149. 10.1016/j.canlet.2010.08.014 20947248 PMC3212431

[B2] Amsberg G. K. Koschmieder S. (2013). Profile of bosutinib and its clinical potential in the treatment of chronic myeloid leukemia. Onco Targets Ther. 6, 99–106. 10.2147/OTT.S19901 23493838 PMC3594007

[B3] Araujo I. M. Abreu S. C. Maron-Gutierrez T. Cruz F. Fujisaki L. Carreira H.. (2010). Bone marrow-derived mononuclear cell therapy in experimental pulmonary and extrapulmonary acute lung injury. Crit. Care Med. 38, 1733–1741. 10.1097/CCM.0b013e3181e796d2 20562701

[B4] Bates J. H. Decramer M. Chartrand D. Zin W. A. Boddener A. Milic-Emili J. (1985). Volume-time profile during relaxed expiration in the normal dog. J. Appl. Physiol. 59, 732–737. 4055563 10.1152/jappl.1985.59.3.732

[B5] Boorsma C. E. Draijer C. Melgert B. N. (2013). Macrophage heterogeneity in respiratory diseases. Mediators Inflamm. 2013:769214. 10.1155/2013/769214 23533311 PMC3600198

[B6] Borges V. M. Falcão H. Leite-Júnior J. H. Alvim L. Teixeira G. P. Russo M.. (2001). Fas ligand triggers pulmonary silicosis. J. Exp. Med. 194, 155–164. 10.1084/jem.194.2.155 11457890 PMC2193452

[B7] Borges V. M. Lopes M. F. Falcão H. Leite-Júnior H. Rocco P. R. Davidson W. F.. (2002). Apoptosis underlies immunopathogenic mechanisms in acute silicosis. Am. J. Respir. Cell Mol. Biol. 27, 78–84. 10.1165/ajrcmb.27.1.4717 12091249

[B8] Bourlier V. Zakaroff-Girard A. Miranville A. De Barros S. Maumus M. Sengenes C.. (2008). Remodeling phenotype of human subcutaneous adipose tissue macrophages. Circulation 117, 806–815. 10.1161/CIRCULATIONAHA.107.724096 18227385

[B9] Chaudhary N. I. Roth G. J. Hilberg F. Müller-Quernheim J. Prasse A. Zissel G. (2007). Inhibition of PDGF, VEGF and FGF signaling attenuates fibrosis. Eur. Respir. J. 29, 976–985. 10.1183/09031936.00152106 17301095

[B10] Chen C. Yang S. Zhang M. Zhang Z. Zhang B. Han D.. (2013). *In vitro* Sirius Red collagen assay measures the pattern shift from soluble to deposited collagen. Adv. Exp. Med. Biol. 765, 47–53. 10.1007/978-1-4614-4989-8_7 22879013

[B11] Cortes J. E. Kantarjian H. M. Brümmendorf T. H. Kim D. W. Turkina A. G. Shen Z. X. (2011). Safety and efficacy of bosutinib (SKI-606) in chronic phase Philadelphia chromosome-positive chronic myeloid leukemia patients with resistance or intolerance to imatinib. Blood 118, 4567–4576. 10.1182/blood-2011-05-355594 21865346 PMC4916618

[B12] Craighead J. E. Kleinerman J. Abraham J. L. Gibbs A. R. Green F. H. Y. Harley R. A. (1988). Diseases associated with exposure to silica and non-fibrous silicate minerals. Arch. Pathol. Lab. Med. 112, 673–720. 2838005

[B13] Cruz F. F. Horta L. F. Maia L. A. Lopes-Pacheco M. da Silva A. B. Morales M. M.. (2016). Dasatinib reduces lung inflammation and fibrosis in acute experimental silicosis. PLoS ONE 11:e0147005. 10.1371/journal.pone.0147005 26789403 PMC4720427

[B14] da Silva A. L. Magalhães R. F. Branco V. C. Silva J. D. Cruz F. F. Marques P. S.. (2016). The tyrosine kinase inhibitor dasatinib reduces lung inflammation and remodelling in experimental allergic asthma. Br. J. Pharmacol. 173, 1236–1247. 10.1111/bph.13430 26989986 PMC5341339

[B15] Davis G. S. Holmes C. E. Pfeiffer L. M. Hemenway D. R. (2001). Lymphocytes, lymphokines, and silicosis. J. Environ. Pathol. Toxicol. Oncol. 20, 53–65. 10.1615/JEnvironPatholToxicolOncol.v20.iSuppl.1.50 11570674

[B16] Dehm S. M. Bonham K. (2004). SRC gene expression in human cancer: the role of transcriptional activation. Biochem. Cell Biol. 82, 263–274. 10.1139/o03-077 15060621

[B17] Di Giuseppe M. Gambelli F. Hoyle G. W. Lungarella G. Studer S. M. Richards T.. (2009). Systemic inhibition of NF-κB activation protects from silicosis. PLoS ONE 4:e5689. 10.1371/journal.pone.0005689 19479048 PMC2682759

[B18] Endo M. Oyadomari S. Terasaki Y. Takeya M. Suga M. Mori M.. (2003). Induction of arginase I and II in bleomycin-induced fibrosis of mouse lung. Am. J. Physiol. Lung Cell. Mol. Physiol. 285, L313–L321. 10.1152/ajplung.00434.2002 12679322

[B19] Faffe D. S. Silva G. H. Kurtz P. M. Negri E. M. Capelozzi V. L. Rocco P. R.. (2001). Lung tissue mechanics and extracellular matrix composition in a murine model of silicosis. J. Appl. Physiol. 90, 1400–1406. 11247940 10.1152/jappl.2001.90.4.1400

[B20] Ferreira T. P. de Arantes A. C. do Nascimento C. V. Olsen P. C. Trentin P. G. Rocco P. R.. (2013). IL-13 immunotoxin accelerates resolution of lung pathological changes triggered by silica particles in mice. J. Immunol. 191, 5220–5229. 10.4049/jimmunol.1203551 24133168

[B21] Finn R. S. Bengala C. Ibrahim N. Roché H. Sparano J. Strauss L. C.. (2011). Dasatinib as a single agent in triple-negative breast cancer: results of an open-label phase 2 study. Clin. Cancer Res. 17, 6905–6913. 10.1158/1078-0432.CCR-11-0288 22028489

[B22] Fubini B. Hubbard A. (2003). Reactive oxygen species (ROS) and reactive nitrogen species (RNS) generation by silica in inflammation and fibrosis. Free Radic. Biol. Med. 34, 1507–1516. 10.1016/S0891-5849(03)00149-7 12788471

[B23] Futosi K. Németh T. Pick R. Vántus T. Walzog B. Mócsai A. (2012). Dasatinib inhibits proinflammatory functions of mature human neutrophils. Blood 119, 4981–4991. 10.1182/blood-2011-07-369041 22411867 PMC3367900

[B24] Garn H. Friedetzky A. Kirchner A. Jäger R. Gemsa D. (2000). Experimental silicosis: a shift to a preferential IFN-gamma-based Th1 response in thoracic lymph nodes. Am. J. Physiol. Lung Cell. Mol. Physiol. 278, L1221–L1230. 10835328 10.1152/ajplung.2000.278.6.L1221

[B25] Goldenberg M. M. (2012). Pharmaceutical approval update. P T. 37, 620–649. 23204815 PMC3498995

[B26] Greenberg M. I. Waksman J. Curtis J. (2007). Silicosis: a review. Dis. Mon. 53, 394–416. 10.1016/j.disamonth.2007.09.020 17976433

[B27] Grimminger F. Schermuly R. T. Ghofrani H. A. (2010). Targeting non-malignant disorders with tyrosine kinase inhibitors. Nat. Rev. Drug Discov. 9, 956–970. 10.1038/nrd3297 21119733

[B28] Hauck C. R. Hsia D. A. Schlaepfer D. D. (2002). The focal adhesion kinase-a regulator of cell migration and invasion. IUBMB Life 53, 115–119. 10.1080/15216540211470 12049193

[B29] Herbert D. R. Hölscher C. Mohrs M. Arendse B. Schwegmann A. Radwanska M.. (2004). Alternative macrophage activation is essential for survival during schistosomiasis and downmodulates T helper 1 responses and immunopathology. Immunity 20, 623–635. 10.1016/S1074-7613(04)00107-4 15142530

[B30] Hopkins A. L. Groom C. R. (2002). The druggable genome. Nat. Rev. Drug Discov. 1, 727–730. 10.1038/nrd892 12209152

[B31] Hu G. Place A. T. Minshall R. D. (2008). Regulation of endothelial permeability by Src kinase signaling: vascular leakage versus transcellular transport of drugs and macromolecules. Chem. Biol. Interact. 171, 177–189. 10.1016/j.cbi.2007.08.006 17897637 PMC3001132

[B32] Hubbard A. K. Mowbray S. Thibodeau M. Giardina C. (2005). Silica-induced inflammatory mediators and pulmonary fibrosis, in Fibrogenesis: Cellular and Molecular Basis ed Mohammed S. R.(Boston, MA: Springer), 199–210.

[B33] Johnson F. M. Gallick G. E. (2007). SRC family nonreceptor tyrosine kinases as molecular targets for cancer therapy. Anticancer Agents Med. Chem. 7, 651–659. 10.2174/187152007784111278 18045060

[B34] Kang J. L. Jung H. J. Lee K. Kim H. R. (2006). Src tyrosine kinases mediate crystalline silica-induced NF-κB activation through tyrosine phosphorylation of IκB-a and p65 NF-κB in RAW 264.7 macrophages. Toxicol. Sci. 90, 470–477. 10.1093/toxsci/kfj096 16431847

[B35] Kawasaki H. (2015). A mechanistic review of silica-induced inhalation toxicity. Inhal. Toxicol. 27, 363–377. 10.3109/08958378.2015.1066905 26194035

[B36] Keller V. A. Brummendorf T. H. (2012). Novel aspects of therapy with the dual Src and Abl kinase inhibitor bosutinib in chronic myeloid leukemia. Expert Rev. Anticancer Ther. 12, 1121–1127. 10.1586/era.12.84 23098112

[B37] Khadaroo R. G. Kapus A. Powers K. A. Cybulsky M. I. Marshall J. C. Rotstein O. D. (2003). Oxidative stress reprograms lipopolysaccharide signaling via Src kinase-dependent pathway in RAW264.7, macrophage cell line. J. Biol. Chem. 278, 47834–47841. 10.1074/jbc.M302660200 12896983

[B38] Khoury H. J. Cortes J. E. Kantarjian H. M. Gambacorti-Passerini C. Baccarani M. Kim D. W. (2012). Bosutinib is active in chronic phase chronic myeloid leukemia after imatinib and dasatinib and/or nilotinib therapy failure. Blood 119, 3403–3412. 10.1182/blood-2011-11-390120 22371878 PMC4916559

[B39] Kluger H. M. Dudek A. Z. McCann C. Ritacco J. Southard N. Jilaveanu L. B.. (2011). A phase 2 trial of dasatinib in advanced melanoma. Cancer 117, 2202–2208. 10.1002/cncr.25766 21523734 PMC3116034

[B40] Kong D. Chen F. Sima N. I. (2015). Inhibition of focal adhesion kinase induces apoptosis in bladder cancer cells via Src and the phosphatidylinositol 3-kinase/Akt pathway. Exp. Ther. Med. 10, 1725–1731. 10.3892/etm.2015.2745 26640543 PMC4665970

[B41] Lagares D. Busnadiego O. García-Fernández R. A. Kapoor M. Liu S. Carter D. E.. (2012). Inhibition of focal adhesion kinase prevents experimental lung fibrosis and myofibroblast formation. Arthritis Rheum. 64, 1653–1664. 10.1002/art.33482 22492165 PMC3338902

[B42] Laney A. S. Attfield M. D. (2010). Coal workers' pneumoconiosis and progressive massive fibrosis are increasingly more prevalent among workers in small underground coal mines in the United States. Occup. Environ. Med. 67, 428–431. 10.1136/oem.2009.050757 20522823

[B43] Lemmon M. A. Schlessinger J. (2010). Cell signaling by receptor tyrosine kinases. Cell 141, 1117–1134. 10.1016/j.cell.2010.06.011 20602996 PMC2914105

[B44] Leung C. C. Yu I. T. Chen W. (2012). Silicosis. Lancet 379, 2008–2018. 10.1016/S0140-6736(12)60235-9 22534002

[B45] Li S. (2008). Src-family kinases in the development and therapy of Philadelphia chromosome-positive chronic myeloid leukemia and acute lymphoblastic leukemia. Leuk. Lymphoma 49, 19–26. 10.1080/10428190701713689 18203007 PMC2430171

[B46] Liu Y. Y. Li L. F. Fu J. Y. Kao K. C. Huang C. C. Chien Y. (2014). Induced pluripotent stem cell therapy ameliorates hyperoxia-augmented ventilator-induced lung injury through suppressing the Src pathway. PLoS ONE 13:e109953 10.1371/journal.pone.0109953 PMC4195701 25310015

[B47] Lopes-Pacheco M. Ventura T. G. de Oliveira H. D. Monção-Ribeiro L. C. Gutfilen B. de Souza S. A. (2014). Infusion of bone marrow mononuclear cells reduces lung fibrosis but not inflammation in the late stages of murine silicosis. PLoS ONE 9:e109982 10.1371/journal.pone.0109982 25299237 PMC4192548

[B48] Lopes-Pacheco M. Xisto D. G. Ornellas F. M. Antunes M. A. Abreu S. C. Rocco P. R. M.. (2013). Repeated administration of bone marrow-derived cells prevents disease progression in experimental silicosis. Cell Physiol. Biochem. 32, 1681–1694. 10.1159/000356603 24356399

[B49] Manning G. Whyte D. B. Martinez R. Hunter T. Sudarsanam S. (2002). The protein kinase complement of the human genome. Science 298, 1912–1934. 10.1126/science.1075762 12471243

[B50] Maron-Gutierrez T. Castiglione R. C. Xisto D. G. Oliveira M. G. Cruz F. F. Peçanha R.. (2011). Bone marrow-derived mononuclear cell therapy attenuates silica-induced lung fibrosis. Eur. Respir. J. 37, 1217–1225. 10.1183/09031936.00205009 20693250

[B51] Misson P. Van den Brûle S. Barbarin V. Lison D. Huaux F. (2004). Markers of macrophage differentiation in experimental silicosis. J. Leukoc. Biol. 76, 926–932. 10.1189/jlb.0104019 15292275

[B52] Mosser D. M. (2003). The many faces of macrophage activation. J. Leukoc. Biol. 73, 209–212. 10.1189/jlb.0602325 12554797

[B53] Okutani D. Lodyga M. Han B. Liu M. (2006). Src protein tyrosine kinase family and acute inflammatory responses. Am. J. Physiol. Lung Cell. Mol. Physiol. 291, L129–L141. 10.1152/ajplung.00261.2005 16581827

[B54] Oliveira G. P. Silva J. D. Marques P. S. Gonçalves-de-Albuquerque C. F. Santos H. L. Vasconcellos A. P.. (2015). The effects of dasatinib in experimental acute respiratory distress syndrome depend on dose and etiology. Cell. Physiol. Biochem. 36, 1644–1658. 10.1159/000430325 26160269

[B55] Overington J. P. Al-Lazikani B. Hopkins A. L. (2006). How many drug targets are there? Nat. Rev. Drug Discov. 5, 993–996. 10.1038/nrd2199 17139284

[B56] Ozanne J. Prescott A. R. Clark K. (2015). The clinically approved drugs dasatinib and bosutinib induce anti-inflammatory macrophages by inhibiting the salt-inducible kinases. Biochem. J. 465, 271–279. 10.1042/BJ20141165 25351958 PMC4286194

[B57] Page T. H. Smolinska M. Gillespie J. Urbaniak A. M. Foxwell B. M. (2009). Tyrosine kinases and inflammatory signaling. Curr. Mol. Med. 9, 69–85. 10.2174/156652409787314507 19199943

[B58] Pesce J. T. Ramalingam T. R. Mentink-Kane M. M. Wilson M. S. El Kasimi K. C. Smith A. M.. (2009). Arginase-1-expressing macrophages suppress Th2 cytokine-driven inflammation and fibrosis. PLoS Pathog. 5:e1000371. 10.1371/journal.ppat.1000371 19360123 PMC2660425

[B59] Portaa C. Rimoldic M. Raesd G. Brysd L. Ghezzie P. Di Liberto D. Dieli F. (2009). Tolerance and M2 (alternative) macrophage polarization are related processes orchestrated by p50 nuclear factor κB. Proc. Natl. Acad. Sci. U.S.A. 106, 14978–14983. 10.1073/pnas.0809784106 19706447 PMC2736429

[B60] Puttini M. Coluccia A. M. Boschelli F. Cleris L. Marchesi E. Donella-Deana A.. (2006). *In vitro* and *in vivo* activity of SKI-606, a novel Src-Abl inhibitor, against imatinib-resistant Bcr-Abl+ neoplastic cells. Cancer Res. 66, 11314–11322. 10.1158/0008-5472.CAN-06-1199 17114238

[B61] Reiske H. R. Zhao J. Han D. C. Cooper L. A. Guan J. L. (2000). Analysis of FAK associated signaling pathways in the regulation of cell cycle progression. FEBS Lett. 486, 275–280. 10.1016/S0014-5793(00)02295-X 11119718

[B62] Remsing Rix L. L. Rix U. Colinge J. Hantschel O. Bennett K. L. Stranzl T.. (2009). Global target profile of the kinase inhibitor bosutinib in primary chronic myeloid leukemia cells. Leukemia 23, 477–485. 10.1038/leu.2008.334 19039322

[B63] Richeldi L. Costabel U. Selman M. Kim D. S. Hansell D. M. Nicholson A. G.. (2011). Efficacy of a tyrosine kinase inhibitor in idiopathic pulmonary fibrosis. N. Engl. J. Med. 365, 1079–1087. 10.1056/NEJMoa1103690 21992121

[B64] Rimal B. Greenberg A. K. Rom W. N. (2005). Basic pathogenetic mechanisms in silicosis: current understanding. Curr. Opin. Pulm. Med. 11, 169–173. 10.1097/01.mcp.0000152998.11335.24 15699791

[B65] Salmond R. J. Filby A. Qureshi I. Caserta S. Zamoyska R. (2009). T-cell receptor proximal signaling via the Src-family kinases, Lck and Fyn, influences T-cell activation, differentiation, and tolerance. Immunol. Rev. 228, 9–22. 10.1111/j.1600-065X.2008.00745.x 19290918

[B66] Setoguchi K. Takeya M. Akaike T. Suga M. Hattori R. Maeda H.. (1996). Expression of inducible nitric oxide synthase and its involvement in pulmonary granulomatous inflammation in rats. Am. J. Pathol. 149, 2005–2022. 8952535 PMC1865352

[B67] Siesser P. M. Meenderink L. M. Ryzhova L. Michael K. E. Dumbauld D. W. García A. J.. (2008). A FAK/Src chimera with gain-of-function properties promotes formation of large peripheral adhesions associated with dynamic actin assembly. Cell Motil. Cytoskeleton 65, 25–39. 10.1002/cm.20241 17922492 PMC2387247

[B68] Suarthana E. Laney A. S. Storey E. Hale J. M. Attfield M. D. (2011). Coal workers' pneumoconiosis in the United States: regional differences 40 years after implementation of the 1969 Federal Coal Mine Health and Safety Act. Occup. Environ. Med. 68, 908–913. 10.1136/oem.2010.063594 21597107

[B69] Summy J. M. Gallick G. E. (2003). Src family kinases in tumor progression and metastasis. Cancer Metastasis Rev. 22, 337–358. 10.1023/A:1023772912750 12884910

[B70] Thakur A. S. Beamer C. A. Migliaccio C. T. Holian A. (2009). Critical of MARCO on crystalline silica-induced pulmonary inflammation. Toxicol. Sci. 108, 462–471. 10.1093/toxsci/kfp011 19151164 PMC2664690

[B71] Thomas S. M. Brugge J. S. (1997). Cellular functions regulated by Src family kinases. Annu. Rev. Cell. Dev. Biol. 13, 513–609. 10.1146/annurev.cellbio.13.1.513 9442882

[B72] Vultur A. Buettner R. Kowolik C. Liang W. Smith D. Boschelli F.. (2008). SKI-606 (bosutinib), a novel Src kinase inhibitor, suppresses migration and invasion of human breast cancer cells. Mol. Cancer Ther. 7, 1185–1194. 10.1158/1535-7163.MCT-08-0126 18483306 PMC2794837

[B73] Wang Y. Wang Y. P. Zheng G. Lee V. W. Ouyang L. Chang D. H.. (2007). *Ex vivo* programmed macrophages ameliorate experimental chronic inflammatory renal disease. Kidney Int. 72, 290–299. 10.1038/sj.ki.5002275 17440493

[B74] Watanabe H. Numata K. Ito T. (2004). Innate immune response in Th1- and Th2-dominant mouse strains. Shock 22, 460–466. 10.1097/01.shk.0000142249.08135.e9 15489639

[B75] Weibel E. R. (1990). Morphometry: stereological theory and practical methods, in Models of Lung Disease Microscopy and Structural Methods, ed Gil J.(New York, NY: Marcel Dekker), 199–247.

[B76] Weng S. Y. Padberg K. Wang X. Y. Kim Y. O. Crosby J. McCaleb M. (2014). P633 Regulation of liver fibrosis by tuning M2 macrophage polarization through IL-4R inhibition. J. Hepatol. 60, S279–S280. 10.1016/S0168-8278(14)60795-9

[B77] Wilson H. M. Walbaum D. Rees A. J. (2004). Macrophages and the kidney. Curr. Opin. Nephrol. Hypertens. 13, 285–290. 10.1097/00041552-200405000-00004 15073486

[B78] Yoshiji H. Kuriyama S. Yoshii J. Ikenaka Y. Noguchi R. Nakatani T.. (2002). Tissue inhibitor of metalloproteinases-1 attenuates spontaneous liver fibrosis resolution in the transgenic mouse. Hepatology 36, 850–860. 10.1053/jhep.2002.35625 12297832

[B79] Zarbock A. Ley K. (2011). Protein tyrosine kinases in neutrophil activation and recruitment. Arch. Biochem. Biophys. 510, 112–119. 10.1016/j.abb.2011.02.009 21338576

[B80] Zhao X. K. Cheng Y. Liang Cheng M. Yu L. Mu M. Li H. (2016). Focal adhesion kinase regulates fibroblast migration via integrin beta-1 and plays a central role in fibrosis. Sci. Rep. 14:19276 10.1038/srep19276 PMC4725867 26763945

